# Observation of Blood Donor-Recipient Malaria Parasitaemia Patterns in a Malaria Endemic Region

**DOI:** 10.1155/2017/7149261

**Published:** 2017-08-24

**Authors:** Jamilu Abdullahi Faruk, Gboye Olufemi Ogunrinde, Aisha Indo Mamman

**Affiliations:** ^1^Paediatrics Haematology-Oncology Unit, Department of Paediatrics, Ahmadu Bello University Teaching Hospital, PMB 06, Shika-Zaria, Kaduna State, Nigeria; ^2^Department of Haematology and Blood Transfusion, Ahmadu Bello University Teaching Hospital, PMB 06, Shika-Zaria, Kaduna State, Nigeria

## Abstract

**Background:**

Asymptomatic malaria parasitaemia has been documented in donor blood in West Africa. However, donated blood is not routinely screened for malaria parasites (MPs). The present study therefore aimed to document the frequency of blood transfusion-induced donor-recipient malaria parasitaemia patterns, in children receiving blood transfusion in a tertiary health-centre.

**Methodology:**

A cross-sectional, observational study involving 140 children receiving blood transfusion was carried out. Blood donor units and patients' blood samples were obtained, for the determination of malaria parasites (MPs). Giemsa staining technique was used to determine the presence of malaria parasitaemia.

**Results:**

Malaria parasites were detected in 7% of donor blood and in 8.3% of the recipients' pretransfusion blood. The incidence of posttransfusion MPs was 3%, but none of these were consistent with blood transfusion-induced malaria, as no child with posttransfusion parasitaemia was transfused with parasitized donor blood. Majority of the blood transfusions (89.4%) had no MPs in either donors or recipients, while 6.8% had MPs in both donors and recipients, with the remaining 3.8% showing MPs in recipients alone.

**Conclusion:**

In conclusion, the incidence of posttransfusion malaria parasitaemia appears low under the prevailing circumstances.

## 1. Introduction

The transmission of malaria through blood transfusion was one of the first recorded transfusion-transmissible infections [1], and the risk of transmitting malaria by blood transfusion is high in malaria endemic countries [2].

According to a World Health Organization (WHO) report, only four transfusion-transmitted infections are tested for in Africa, namely, human immune-deficiency virus (HIV), hepatitis B virus (HBV), hepatitis C virus (HCV), and syphilis [3], malaria not being among them. In Nigeria, donated blood is also not routinely screened for malaria parasites before transfusion [4], but asymptomatic malaria parasitaemia has been demonstrated in 11% (Jos) [5], 6.8% (Zaria) [6], 16.4% (Lagos) [7], 40.9% (Abakaliki) [8], 30.2% (Nnewi) [9], 40% (Benin City) [10], and 41% (Ibadan) [11] of blood donors. As a result of the high rate of blood transfusions in our environment [12], the risk of blood transfusion-induced malaria (BTM) assumes significant health importance. Ibhanesebhor [13] documented a 13% prevalence of blood transfusion-induced malaria among babies in Benin. Consequently, the administration of antimalarials to recipients of blood transfusion has been suggested [8].

The cost of screening blood routinely for MPs is enormous [14], and a policy excluding MPs-positive blood donors would lead to wastage of potentially life-saving blood products [15]. This will further deplete an already overstretched blood supply system in developing countries [16]. Furthermore, the risk of contracting malaria from natural infection is still high in these endemic areas. Thus, blood is not routinely screened for MPs in malaria endemic areas [16]. This practice, however, exposes recipients of blood transfusion to the risk of contracting malaria infection through this route.

Many studies have documented the prevalence of MPs among blood donors and therefore their potential to cause BTM. However, few studies have demonstrated the actual incidence of BTM in blood recipients, especially among children. And fewer studies have described the proportion of transfusions that are free of BTM from a malaria endemic area.

In view of the above facts, there is a need to carry out an observational study on blood transfusion-induced malaria in a malaria endemic area, to document the patterns of MPs between donors and recipients of blood transfusions. This study attempts to determine the burden of transfusion malaria, in children admitted to Ahmadu Bello University Teaching Hospital (ABUTH), Shika-Zaria, Nigeria.

## 2. Patients and Methods

The study was conducted at the paediatric medical and surgical wards of ABUTH, Shika-Zaria, a tertiary health facility for many northern states of Nigeria. The study was descriptive and cross-sectional, spanning from November 2011 to May 2012, which corresponds to the off-peak or low-malaria transmission season. Children aged 0–12 years that were admitted into the aforementioned wards of ABUTH, who required and subsequently received a blood transfusion during the study period, were enrolled. Patients who were to receive a second blood transfusion within 48 hours of a transfusion were excluded from the study. Approval was obtained from the Scientific and Ethical Committee of ABUTH before commencement of the study. Informed written consent was obtained from parent(s) or guardian(s) of each patient before enrollment into the study. Verbal assent was also obtained from children old enough to understand (seven years and older). Those who consented were recruited consecutively, until the desired quota sample size was obtained.

Patients that fulfilled the inclusion criteria were recruited consecutively, until the required sample size was obtained. Quota sampling was employed in order to capture the different paediatric age groups requiring blood transfusion. Thus, 28 neonates [0 to 28 days], 28 infants [29 days to 11 months (mo)], 28 toddlers [1 yr to 2 yr 11 mo], 28 preschoolers [3 yr to 5 yr 11 mo], and 28 school-age children [6 yr to 12 yr 11 mo] were recruited.

Finger prick blood samples were obtained in capillary tubes before and after blood transfusion for preparation of thin and thick blood films for the detection of MPs and parasite densities, using Giemsa staining technique [17]. One milliliter of donor blood unit was collected from the blood bag, through a blood giving set, before it was connected to the recipients, for MPs detection using the same technique. The first blood sample for patient's MPs was taken immediately before the transfusion and the patient's second blood sample was taken within 24–48 hours after the transfusion.

A positive result in the posttransfusion blood film of a recipient was termed posttransfusion malaria parasitaemia, and conversely absence of MPs in the posttransfusion film was termed no posttransfusion MPs. Where the donor had positive MPs, but recipient had a negative pretransfusion blood film, then a positive posttransfusion film would be suggestive of blood transfusion-induced MPs. On the other hand, positive donor MPs, with a negative pretransfusion and negative posttransfusion film, implied no blood transfusion-induced MPs. If, however, there was a negative result in the donor, but recipient had a positive result in the posttransfusion film, with or without a positive pretransfusion film, then it was termed posttransfusion malaria. Presence of positive results in all the three cohorts of specimens (donor and recipient pretransfusion and posttransfusion samples) was also termed posttransfusion MPs.

Data obtained from the study were analyzed using the statistical software EPI-info 3.5.3. A *p* value of less than 0.05 was considered statistically significant in comparative analysis.

## 3. Results

Out of the 140 subjects recruited into the study, 132 completed their blood transfusions and had the posttransfusion blood film examined. There were 78 males and 54 females, with a ratio of 1.4 : 1. These comprised 26 neonates (mean age 7.9 ± 4.7 days); 27 infants (mean age 6.4 ± 3.0 months); 25 toddlers (mean age 22.1 ± 7.7 months); 27 preschoolers (mean age 51.6 ± 10.7 months); 27 school-aged subjects (mean age 8.4 ± 1.9 years).

A total of 138 blood donor units were used for the study participants. Out of these blood donor units, nine units were infected with MPs, giving a malaria parasitaemia prevalence of 7% among blood donors. There were 11 positive MPs in the pretransfusion samples among the 132 subjects, giving a pretransfusion malaria parasitaemia prevalence of 8.3%. On the other hand, four out of the 132 recipients had positive MPs in their posttransfusion samples at the end of the study. This gave a posttransfusion malaria parasitaemia incidence of 3%.

*P. falciparum* trophozoites were demonstrated in all samples that were positive for MPs. No mixed infections were detected.

The positivity rate was not significantly different in the donor blood compared to the recipients pre- and posttransfusion samples (chi-square = 3.42, degrees of freedom 2, *p* value = 0.18). The four cases of posttransfusion malaria parasitaemia were recorded in one male neonate, two female preschoolers, and one male school-aged.

Table 1 describes the patterns encountered of the distribution of parasitaemia between donors and recipients in the study. The predominant pattern seen was where there were no MPs in donors and no MPs in recipients before transfusion and after transfusion. This occurred in 89.4% of transfusions (scenario 1). In 6.8% of transfusions, the donor was positive for MPs and there were coincident positive MPs in recipients before transfusion, but after transfusion there were no MPs detected (scenario 2). This scenario accounted for the entire donor MPs in the study.

It was further observed from the table that, in scenario 3, a total 2.3% of the transfusions had no MPs in the donor, and no MPs were detected before transfusion, but the posttransfusion sample was positive for MPs. The remaining two transfusions revealed no MPs in the donor blood, with positive MPs in the recipients before transfusion, but one had positive MPs and the other had negative MPs after transfusion (scenarios 4 and 5, resp.). Each of these accounted for 0.8% of the study.

Interestingly, no scenario was encountered where a donor had MPs, and the recipient developed MPs in the posttransfusion sample. So, there was no case of blood transfusion MPs detected in the study.

As shown in Table 2, the parasite density range was 18/*μ*l–550/*μ*l in the donor group, while it was relatively higher at 28/*μ*l–5,080/*μ*l in the pretransfusion group. On the other hand, the parasite density range was 74/*μ*l–100/*μ*l in the posttransfusion group. Among the cohorts with positive MPs in both donor and pretransfusion samples, the recipients had higher parasite densities before transfusion compared to their donors in three transfusions (serial numbers (1)–(3) in Table 2), while the donors had higher parasitaemia compared to their corresponding recipients in six transfusions (serial numbers (4)–(9) in Table 2). It can also be observed from the table that, in serial number 10, the recipient had a higher parasite density in the posttransfusion compared to the pretransfusion sample even though no MPs were transfused from the donor.

The geometric mean parasite densities showed that the average parasitaemia in donors and pretransfusion recipients was similar (102/*μ*l and 100/*μ*l, resp.) and relatively higher than the average posttransfusion parasitaemia (83/*μ*l).

Using the “plus” system of estimating density of MPs, seven of nine (77.8%) of donors had +, and the other 22.2% had densities of ++ each. No donor had +++ or ++++. In the pretransfusion group, nine (81.8%) had +, one (9.1%) had ++, and the remaining one (9.1%) had +++. All the posttransfusion MPs had densities of +.

## 4. Discussion

The study detected the presence of malaria parasites in donated blood units and the recipients of blood in their pretransfusion and posttransfusion samples. As demonstrated by microscopy, the prevalence of malaria parasitaemia in blood donor units of 7% obtained in this study is low, although not insignificant. This low prevalence may be accounted for by the study period which was in the dry season, with low transmission rates of malaria. The timing of the study from November to May, and the duration of seven months, did not cover the peak rainfall period of April to October. This timing could have influenced the prevalence of malaria in the population. Malaria transmission is favoured by the availability of stagnant surface waters that are abundant during the rainy season; these act as breeding sites for the vector mosquitoes of the infection. The studies from Abakaliki [8] and Nnewi [9] which were conducted during the rainy season had higher parasitaemia levels, compared to the studies from Jos [5] and Zaria [6] which were conducted during the dry season. This disparity in the level of parasitaemia is further buttressed by the systemic review of Owusu-Ofori et al. [18] whereby the studies done during high malaria transmission seasons had relatively higher donor parasitaemia compared to those done during low-malaria transmission seasons.

Another factor influencing the study's low donor parasitaemia could be the relatively small sample size of 132, although statistically it is adequate to demonstrate the desired parameter. However, a larger sample size would have increased the power or accuracy of the study, and the obtained prevalence rate might have been higher. This effect of sample size can be seen in the studies from Abakaliki [8] and Nnewi [9] that had large sample sizes and concomitant high prevalence of 40.9% and 30.2%. respectively. On the other hand, the studies from Jos [5] and Zaria [6] with small sample sizes had lower prevalence of 11% and 6.8%, respectively.

The prevalence of MPs in blood donor units in the study of 7% is closer to the levels found by the workers in Jos [5] and Zaria [6], all of which incidentally were from northern Nigeria. They are much lower than the prevalence rates obtained from the southern part of the country (Abakaliki [8] and Nnewi [9]), despite the fact that all the workers used similar Giemsa staining techniques, and all the studies were conducted in consecutively recruited prospective blood donors, apart from the study from Nnewi, which employed systematic random sampling. These differences between the north and south might be accounted for by the difference in malaria transmission across the savannah region of the north which is mesoendemic, with seasonal transmission, and the rain forest region of the south which is hyperendemic, with perennial transmission. Other factors explaining this difference include the relatively larger sample sizes and longer study periods which coincided with the rainy seasons in the studies from the south.

Interestingly, the prevalence of malaria parasitaemia in the pretransfusion samples was 8.3%, similar to the 7% prevalence level in the blood donor units. This similarity probably reflects the risks or chances of malaria infection in the general population, and statistically it may suggest that the donors do not pose additional risks to the recipients since their malaria experience is the same. However, this assertion may not be accurate in real life since there will always be a probability that a blood unit containing parasites may be transfused to a recipient hitherto negative for MPs. Again, this assumption of low potential risk will not hold true if the recipients were from a non-malaria endemic region, where the recipients' level of parasitaemia is likely to be naught.

It is difficult to compare the level of MPs in pretransfusion patients of this study with other studies with a similar methodology to this study, since none declared the level of MPs in the pretransfusion samples of their patients. The study of Ali et al. [19] from Sudan excluded recipients with positive MPs before transfusion from their study, so it is not possible to say what proportion of blood recipients harbor MPs prior to their transfusions from that study. Knowledge of this proportion would have enabled one to deduce whether recipients of blood are at a higher risk of natural mosquito-borne malaria infection, if they had a higher prevalence of MPs before transfusion compared to their corresponding donors. Indeed, studies unbiased to transfusion malaria have documented very high pretransfusion parasitaemia of 76–83%, among children requiring emergency blood transfusions in malaria endemic regions [20, 21].

The prevalence of malaria parasitaemia in the posttransfusion samples of the study was 3%, which is quite lower than the donor or the pretransfusion levels. There was no readily discernible explanation, but possibility of confounders like the use of antimalarials cannot be ruled out. Based on microscopy technique, no case of blood transfusion malaria occurred in this study, despite the four cases of posttransfusion malaria seen. None of the 132 patients that completed the study fulfilled the criteria, since none of the participants had a negative pretransfusion sample and received a MPs-positive donor blood unit and then subsequently developed positive MPs after transfusion. When compared with the study by Ali et al. [19] where the posttransfusion MPs prevalence was 3.5%, 12 out of the 14 cases of posttransfusion MPs were all due to infected donor blood. Only two of the posttransfusion MPs were not due to infected donor blood, which resembles the scenario in this study. More importantly, as depicted in scenarios 3 and 4, the source of posttransfusion MPs could arise directly from the recipient's pretransfusion status or may not be obvious from either the donor or pretransfusion state; these groups of recipients may be assumed to be in the incubation period of a natural malaria infection, wherein the MPs become detectable and coincident with the completion of the blood transfusion.

The finding of a zero incidence of blood transfusion-induced malaria is not strange. The study by Onankpa et al. [22] from Sokoto found no case of transfusion malaria from 162 neonates, even though other forms of malaria were found in 96% of the subjects in that study. More recent studies utilizing molecular parasite detection techniques also found low levels of posttransfusion MPs, whether or not deliberate attempts at eradicating MPs were made [23–25].

The predominant scenario of parasite distribution between donor-recipient pairs found in this study was that of negative MPs in donor and pretransfusion and posttransfusion samples (89.4%). Incidentally, all donor units that were positive for MPs in this study were also transfused to recipients with positive pretransfusion MPs, even though none of these had positive MPs after transfusion. While all the “permutations and combinations” of the donor and recipient parasitaemia were entirely based on chance occurrence, the patterns observed may also result from the inability of microscopy to detect very low levels of parasitaemia (<5/*μ*l) which would otherwise convert some negative specimens to positive. Either way, it further shows that the potential risk and occurrence of blood transfusion MPs are very low under the prevailing circumstances. The study by Ali et al. [19] also had a predominant occurrence of scenario one (96.5%), due to the exclusion of patients with positive pretransfusion MPs. So, scenarios two, four, and five were not seen in that study. The other scenario common to both studies was that where donor and pretransfusion samples had no MPs, but the recipients developed MPs after transfusion (scenario three). This scenario implies that not all posttransfusion MPs are blood-induced; the recipients might just have had natural mosquito acquired MPs in the exoerythrocytic stages during the pretransfusion tests. The study by Owusu-Ofori et al. [23] has clearly validated this assertion using molecular techniques.

The average parasite densities obtained in the study were low in the donor blood units. These low levels of parasitaemia in the asymptomatic donors correspond to findings by Ali et al. [19], Ikeh and Okeke [5], and Uneke et al. [8], even though the studies used different class intervals for the parasite densities. This finding may support further why blood transfusion malaria is not common in malaria endemic regions; when there is low parasite density, the fraction of parasites transfused may be negligible especially when only a small portion of the blood unit is utilized. Many blood transfusions in paediatric practice utilize small aliquots of donated blood, as it was found in this study.

## 5. Conclusions

From this study, it can be concluded that malaria parasitaemia is as common in asymptomatic donors as in the recipients of blood transfusion, the incidence of posttransfusion malaria parasitaemia is low in our environment, and blood transfusion-induced malaria is much less common during the dry low-malaria transmission season. The majority of blood transfusions do not contain or transmit malaria parasites during the low-malaria transmission season. And in transfusions that harbor MPs, the density of parasites is lower in asymptomatic blood donors than in recipients of blood transfusions.

## Figures and Tables

**Table 1:** Scenarios of the distribution of donor-recipient malaria parasitaemia among children receiving blood transfusion at ABUTH, Zaria (*n* = 132).

Group	Scenario				
	1	2	3	4	5
Donor unit	MPs−	MPs+	MPs−	MPs−	MPs−
Pretransfusion	MPs−	MPs+	MPs−	MPs+	MPs+
Posttransfusion	MPs−	MPs−	MPs+	MPs+	MPs−
Tally (%)	118 (89.4)	9 (6.8)	3 (2.3)	1 (0.8)	1 (0.8)

MPs−: negative malaria parasites; MPs+: positive malaria parasites.

**Table 2:** Malaria parasite densities in donor-recipient pairs at ABUTH, Zaria (*n* = 14).

Serial number	Parasite densities (/*µ*l)		
	Donor unit	Pretransfusion	Posttransfusion
(1)	172	5,080	MP−
(2)	80	193	MP−
(3)	18	50	MP−
(4)	550	150	MP−
(5)	150	40	MP−
(6)	147	120	MP−
(7)	88	35	MP−
(8)	75	44	MP−
(9)	60	28	MP−
(10)	MP−	63	75
(11)	MP−	MP−	74
(12)	MP−	MP−	100
(13)	MP−	MP−	86
(14)	MP−	100	MP−

MP−: negative malaria parasites.
